# Algae Polyphenolic Compounds and Modern Antibacterial Strategies: Current Achievements and Immediate Prospects

**DOI:** 10.3390/biomedicines8090342

**Published:** 2020-09-11

**Authors:** Natalya N. Besednova, Boris G. Andryukov, Tatyana S. Zaporozhets, Sergey P. Kryzhanovsky, Tatyana A. Kuznetsova, Ludmila N. Fedyanina, Ilona D. Makarenkova, Tatyana N. Zvyagintseva

**Affiliations:** 1 Somov Research Institute of Epidemiology and Microbiology, 690087 Vladivostok, Russia; andrukov_bg@mail.ru (B.G.A.); niiem_vl@mail.ru (T.S.Z.); takuznets@mail.ru (T.A.K.); Ilona_m@mail.ru (I.D.M.); 2 School of Biomedicine, Far Eastern Federal University (FEFU), 690091 Vladivostok, Russia; fedyanina.ln@dvfu.ru; 3 Medical Association of the Far Eastern Branch of the Russian Academy of Sciences, 690022 Vladivostok, Russia; priemmodvoran@mail.ru; 4 Elyakov Pacific Institute of Bioorganic Chemistry, Far Eastern Branch of the Russian Academy of Sciences, 690022 Vladivostok, Russia; zvyag@piboc.dvo.ru

**Keywords:** polyphenols (PP), phlorotannins (PT), marine algae, antibiofilm properties, antibacterial properties, drug resistance of microorganisms

## Abstract

The increasing drug resistance of pathogenic microorganisms raises concern worldwide and necessitates the search for new natural compounds with antibacterial properties. Marine algae are considered a natural and attractive biotechnological source of novel antibiotics. The high antimicrobial activity of their polyphenolic compounds is a promising basis for designing innovative pharmaceuticals. They can become both a serious alternative to traditional antimicrobial agents and an effective supplement to antibiotic therapy. The present review summarizes the results of numerous studies on polyphenols from algae and the range of biological activities that determine their biomedical significance. The main focus is put on a group of the polyphenolic metabolites referred to as phlorotannins and, particularly, on their structural diversity and mechanisms of antimicrobial effects. Brown algae are an almost inexhaustible resource with a high biotechnological potential for obtaining these polyfunctional compounds. An opinion is expressed that the effectiveness of the antibacterial activity of phlorotannins depends on the methods of their extraction aimed at preserving the phenolic structure. The use of modern analytical tools opens up a broad range of opportunities for studying the metabolic pathways of phlorotannins and identifying their structural and functional relationships. The high antimicrobial activity of phlorotannins against both Gram-positive and Gram-negative bacteria provides a promising framework for creating novel drugs to be used in the treatment and prevention of infectious diseases.

## 1. Introduction

Antimicrobials are essential medicines used for the treatment of human and animal diseases. However, the constantly emerging new and expanding ranges of long-existing pathogens that have resistance to antimicrobial drugs in the world’s population are becoming an increasing concern worldwide [1,2]. Antimicrobial drug resistance impedes effective prevention and treatment of a growing number of infections caused by bacteria, parasites, viruses, and fungi. Antibiotic resistance is a natural phenomenon, but the uncontrolled use of these drugs by humans and their inappropriate administration to animals accelerate this process [3]. Treatment of a greater number of infectious diseases (such as pneumonia, tuberculosis, gonorrhea, salmonellosis, etc.) becomes a challenge due to the decreasing effectiveness of antibiotics. Drug resistance results in a longer hospital stay, increased economic costs for health care, and increased mortality [4].

In this regard, there is an urgent need to develop new antimicrobial and non-toxic compounds with improved pharmacological characteristics and a different mechanism of action, to which microorganisms would not develop resistance.

It should be noted that the fewest candidates for medicinal agents have been found among natural objects to date, especially among aquatic organisms, despite the large number of works published in the literature for several decades, considering the antibacterial, antiviral, and antifungal activity of metabolites derived from marine algae and invertebrates.

## 2. General Characteristics of Polyphenolic Compounds from Marine Algae

Algae are a unique raw material for the production of a number of substances with a wide range of useful properties. Their composition is characterized by the specific content of minerals, pigments, lipids, polyphenols, proteins, amino acids, cellulose, polysaccharides, etc.

One of the most significant groups of compounds that determine the biomedical importance of marine algae is polyphenols (PP). Algae accumulate large amounts of these compounds, including, in particular, phloroglucinol and its polymers, phlorotannins (PT) [5,6]. Currently, scientists from many countries are showing increasing interest in these compounds, which is manifested as an increase in the number of publications on problems related to their biological activity worldwide [7,8,9].

The largest proportion of phenolic compounds in green and red algae is represented by bromophenols, phenolic acids, and flavonoids. Phlorotannins are found mainly in brown algae and, in lesser amounts, in red algae (1.8–3.2%); even fewer polyphenols are contained in green algae [10].

Phlorotannins of algae are a heterogeneous, least studied group of molecules, differing in structure and degree of polymerization [11,12]. Like tannins of terrestrial plants, these phenolic compounds are highly soluble in water, strongly bound to proteins, polysaccharides, and other biopolymers, chelate divalent metals, and have a polymer structure. These compounds are composed of phloroglucinol monomeric units (1,3,5-trihydroxybenzene), from which more than 700 natural variations have been obtained and are used in various industries [13]. The parental molecule (1,3,5-trihydroxybenzene) of phloroglucinol is isolated from many sources, including phlorotannins derived from brown algae. Largest amounts of PT are accumulated in *Fucus* algae, reaching 3–12% of dry weight [8,14,15,16,17].

Unlike tannins, PT has a wider molecular weight range, from 126 Da to 650 kDa, but, most commonly, the molecular weight of these biopolymers ranges from 10 to 100 kDa. The concentration of PP in algae varies significantly depending on the size, species, geographic region of growth, age, tissue type, water salinity, season, amount of nutrients, light intensity, water temperature, and extraction method [10,18,19,20]. In some species, their content reaches 20% of weight, while in other algae, their content is close to zero. The typical profile of PT from brown algae with antimicrobial activity is mainly represented by phloroglucinol, eckol, and dieckol [21,22].

Almost 90% of the total amount of PT is found in a free state in membrane-bound vesicles referred to as physodes. The rest of the PP compounds are contained in the cell wall of algae, where they are bound in a complex with alginic acid by covalent ester and hemiacetal bonds and act as a structural component, regulating osmotic pressure [23]. The large differences in molecular weight and isomerization levels make it difficult to characterize polyphenols [24,25]. There is also a lack of information on endogenous digestion and microbial catabolism of PP in algae. It is known that about 90–95% of dietary PPs reach the intestine without changes [26], where their biotransformation and metabolism occur, resulting in the formation of low-molecular-weight (LMW) compounds with lower chemical heterogeneity than that in the original PP [27,28].

Polyphenolic compounds extracted from seaweeds are bioavailable. PPs can be absorbed either directly in the upper digestive tract unchanged or in the lower intestine after being modified by bacteria present there [29].

Some PTs can be found in algae in a sulfated or halogenated state [21]. Their biosynthesis is carried out through the acetate-malonate pathway in the Golgi apparatus in the perinuclear region of the cell. They are usually not secreted, and they can only be extracted through cell destruction.

**Figure 1:**
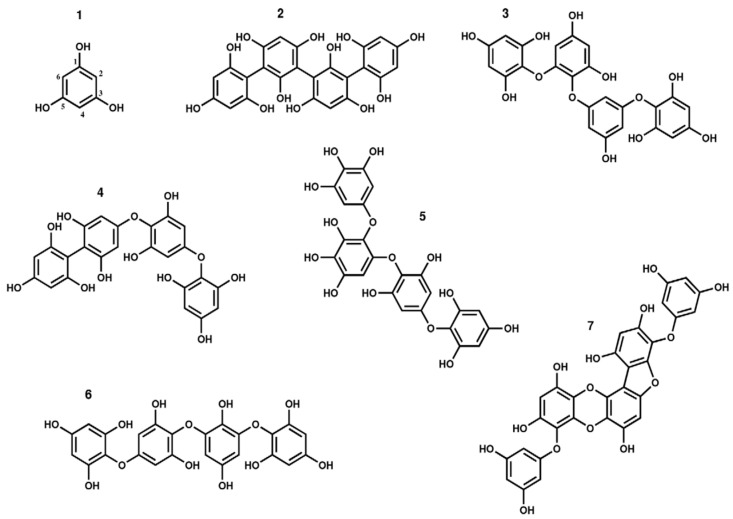
Chemical structures of different types of phlorotannins. Phloroglucinol (**1**), Tetrafucol A (**2**), Tetraphlorethol B (**3**), Fucodiphlorethol A (**4**), Tetrafuhalol A (**5**), Tetraisofuhalol (**6**), and Phlorofucofuroeckol (**7**).

The main challenge in the extraction of PP arises from the strong complexes formed by these compounds with polysaccharides, which are the main components of the cell wall, including PTs [30,31]. In terms of their structure and polymeric properties, PTs represent an extensive group of molecules that differ in the characteristics of bonds between phloroglucinol units and in the number of hydroxyl groups (Figure 1).

Terrestrial plants produce tannins consisting of only 3–4 phenolic rings, while algal PTs consist of eight rings. Phenolic rings act as electron traps for free radicals [32], and, therefore, phlorotannins exhibit very pronounced antioxidant properties. A large number of hydroxyl groups allow PT to produce more hydrogen peroxide, which is toxic to bacteria under aerobic conditions [33]. For example, the PTs from the alga *Eisenia bicyclis* have 10-fold more antioxidant activity than ascorbic acid.

Depending on the type of bond between monomers, phlorotannins are divided into four subclasses: phlorethols and fuhalols; fucols; fucophlorethols; eckols and carmalols [34]. Other authors [21] divide PTs into six groups; it is also suggested to distinguish groups of linear and branched PTs in subclasses (Figure 1).

The pharmacological significance of PPs is associated with their structure, especially with the degree of polymerization. These biologically active compounds have been reported to exhibit anti-inflammatory [35], antiallergic [36], antiviral [37], antitumor [38], antioxidant [35], antidiabetic, and radioprotective effects [39].

As for the relationship between the antioxidant activity and the value of the molecular weight of the compounds, available data are contradictory, and this issue is still under study [40,41,42].

## 3. Obtaining Polyphenols from Marine Algae

Traditionally, extraction with organic solvents is used to extract polyphenols from seaweed (solid-liquid extraction). As extractants, polar solvents, such as ethanol, methanol, acetone, are used, as well as their mixtures with water in various ratios [10,11,43,44,45,46]. The most common reagent for isolating phlorotannins is aqueous solutions of acetone or ethanol. Extraction is usually carried out at a temperature not exceeding 52 °C, since at temperatures above 92 °C, degradation of polyphenolic compounds occurs [10,11].

As is known from the literature, the total content of phenols largely depends on the type of algae [10,11]. Thus, the maximum amount (192.8 ± 3.3 mg/g Gallic acid equivalents, GAE) of polyphenols from *Eisenia bicyclys*, as well as from other types of brown and red algae, is extracted with distilled water and a mixture of water, acetic acid, and methanol (60:69:1 about/in). From *Saccharina japonica*, the highest yield of polyphenols (14.9 ± 0.1 mg/g GAE) is observed with extraction with 80% methanol, and from the alga *Undaria pinnatifida*, with extraction with 100% methanol (8.4 ± 0.2 mg/g GAE) [10].

According to the materials of N.M. Aminina et al. [44], the degree of extraction of PP from *Fucus evanescens* is higher when an aqueous solution of ethanol is used as a solvent, and distilled water is used for extraction from *S. japonica* and *Anfeltia tobuchiensis*. Ethanol extracts of algae from the genera *Agarum*, *Thalassiophyllum*, *Fucus*, *Stephanocystis*, and *Arthrothamnus* show the maximum effect in terms of the sum of PP and antioxidant activity. Promising sources of PP with high antioxidant activity, including PT, *F. evanescens*, *Thalassiophyllum clathrus*, and *Stephanocystis crassipes,* are also included [43,44]. Most authors report that methanol extraction provides the maximum PT yield when using organic solvents [45,46].

Ethyl acetate extracts of other algae, for example, *Ecklonia stolonifera* and *Ecklonia cava*, are also active against methicillin-resistant *Staphylococcus aureus* (MRSA), while diecol is isolated from the extract of the first alga [36,47].

As shown by Li et al. [48], the highest yield of phlorotannin (88.48 ± 0.30 mg PGE/100 mg of extract) is obtained by acetone extraction from algae (PGE means phloroglucinol equivalents). Such extracts are also characterized by the highest antioxidant activity. In recent years, the results of the most efficient production of algae extracts using different extractants and extraction methods have been reported in numerous studies [6,7,40,43].

Solid-liquid extraction methods are widely used for the isolation of phlorotannins from algae; however, they have a number of significant disadvantages, such as low selectivity of the target component, long extraction times to achieve high yields of the polyphenol fraction, and the need to purify the extract from large volumes of organic solvents [10,11,43]. It has been found that during the extraction of polyphenols by classical methods, high-molecular phlorotannins associated with the cell wall remain in algae [44,45].

More efficient methods for the isolation of polyphenols from plant materials can be ultrasonic, microwave, enzymatic extraction, liquid extraction under pressure, and supercritical fluid extraction. The main advantage of these methods is an increase in the yield of the extract in the absence of the destruction of thermolabile compounds [11,43,44,46,47].

The advantage of enzymatic extraction, which has also proven to be effective, is that this method allows the destruction of the algal cell wall and increases the PT yield. For example, the treatment of the alga *Sargassum polycystum* with the Protamex^®^ preparation has made it possible to reach a PT yield of 38.1 ± 6.8% of dry material. Solid-liquid extraction provides a yield of 3–15%; enzymatic extraction, 21–38% [46].

In the process of ultrasonic extraction, mass transfer is activated by destroying the cell walls of plants, which increases the release of high-molecular phlorotannins [43]. The advantage of microwave extraction is a high yield of polyphenolic compounds from plant objects while reducing the extraction time and the amount of solvent [43,44,46]. High-pressure liquid extraction significantly reduces polyphenol recovery time [10,11,43]. Thus, at present, there are a large number of extraction methods that ensure the effective extraction of phlorotannins from algae.

To purify extracts, chromatographic methods are used [46]. Modern methods of identification, quantification, and structural analysis of PTs are based on nuclear magnetic resonance spectroscopy and chromatography-mass spectrometry using various ionization techniques [47]. These methods are described in sufficient detail in numerous works [11,12,18,48].

The major challenge in polyphenol extraction arises from the presence of polysaccharide complexes as the main component of the algal cell wall, while PTs are included in the cell wall [30], being covalently bound to polysaccharides [23] and proteins [49].

## 4. Interaction of Algae Polyphenols with Gram-Positive and Gram-Negative Bacteria

PTs have a high antibacterial activity [31]. At the same time, the sensitivity of Gram-positive and Gram-negative bacteria to these compounds differs: Gram-negative bacteria are less sensitive than Gram-positive ones [50]. PTs can bind to bacterial proteins, such as enzymes, cell membrane proteins, and induce cell lysis [51]. Aromatic rings and OH-groups of phloroglucinol units bind to -NH-groups of bacterial proteins through H-bonds and hydrophobic interactions [52,53].

A higher minimum inhibitory concentration (MIC) is required for penetrating into cells of the Gram-negative *Vibrio parahaemolyticus* compared to the MIC for the Gram-positive methicillin-resistant *Staphylococcus aureus*. This applies to most antibacterial drugs due to the physiological differences in the outer membrane of Gram-positive and Gram-negative bacteria [54,55]. The outer membrane of Gram-negative bacteria is a barrier to many substances, including antibiotics. Nevertheless, algal PT still acts on Gram-negative microorganisms, causing their death.

Thus, Wei et al. [56] reported that low-molecular-weight PTs from the alga *Sargassum thunbergii* damage cell membranes and cell walls of *V. parahaemolyticus*, causing the disturbance of membrane permeability and leakage of intracellular bacterial components. In this case, the cell loses intracellular potassium, nucleic acids, and phosphates [57,58]. The high sensitivity of *Salmonella* sp. to the acetone extract of the green alga *Cladophora socialis*, the main component of which is phenolic compounds, was reported by N. Moubayed with co-authors [51].

An increase in the lipophilic properties of phenolic compounds enhances their antimicrobial activity, promoting their interaction with the cell membrane, causing irreversible changes in cytoplasmic membrane and coagulation of cell contents, including cellular enzymes [57,58]. In case Gram-positive bacteria are exposed to PT, intracellular pH modification and transformations in their energy generation system (ATP) occur [59].

Algal PTs damage and kill both aerobic and anaerobic bacteria. A. Hierholtzer et al. [60] investigated the effect of a crude PT fraction from the marine brown alga *Laminaria digitata* on mixed anaerobic microbial cultures. A transmission electron microscopy (TEM) of anaerobic microorganisms in control preparations (without exposure to PT) shows microorganisms with the normal cell morphology and smooth structure of the cell membrane. After exposure to preparations with phloroglucinol, cells have numerous spore-like structures and disrupted membranes. Endospores are known to appear in microorganisms in adverse habitat conditions [60,61]. Cells exposed to phloroglucinol show signs of membrane dysfunction with the detachment of the cytoplasmic membrane from the cell wall.

PTs cause a stronger inhibition of microorganisms than phloroglucinol (unpolymerized phlorotannin monomer). Thus, the bactericidal activity of PTs depends on the level of their polymerization, which was noted by Nagayama et al. [62].

## 5. Interaction of Algal Polyphenols with Methicillin-Resistant *S. Aureus*

In the course of evolution, bacteria have developed a variety of mechanisms of drug resistance, such as the ability to modify the antibiotic binding site, produce enzymes that can destroy or change the structure of antibiotic, cause mutations in genes encoding transport proteins, leading to disturbance of cell wall permeability, etc.

The methicillin-resistant *S. aureus* (MRSA) is the most commonly occurring multidrug-resistant causative agent of nosocomial infection in Europe. This pathogen is an etiological factor in such dangerous diseases as sepsis, pneumonia, endocarditis, erysipelas, osteomyelitis, and foodborne diseases [63]. Infections caused by this microorganism are characterized by severe course, epidemic spread, often become fatal, accompanied by significant economic damage. There are currently no other effective antibiotics against MRSA than vancomycin, teicoplanin, and arbekacin. However, with the increasing use of vancomycin, the vancomycin-intermediate and vancomycin-resistant variants of the bacterium have already emerged [64].

MRSA first appeared in the late 1970s [65]. The widespread use of methicillin, having resistance to penicillinase, has led to the emergence of this variant of *S. aureus* [66], which also exhibits multidrug resistance [67].

Thus, the development of new drugs with mechanisms of action differing from those of antibiotics, which would be harmless and effective in the treatment of infections caused by MRSA, is an urgent healthcare issue. For this purpose, seaweed extracts have been widely screened.

S. Jegan et al. [18] studied the efficiency of extracts from the algae *Padina tetrastromatica* and *Padina gymnosporia* belonging to the class of brown algae (*Phaeophyceae*). Extracts with increasing polarities (hexane, chloroform, ethyl acetate, and methanol), as well as two standard strains and three clinical isolates of *S. aureus,* are taken for experiments. All extracts have shown significant antibacterial activity against staphylococcus. There are no differences in sensitivity between the standard and clinical strains. The average zone of inhibition of bacterial growth ranges from 7.1 to 26.5 mm, with the positive control of methicillin being from 7.1 to 9.0 mm.

The lowest MIC (62.5 μg/mL) and minimum bactericidal concentration (MBC, 125 μg/mL) values are observed for the ethyl acetate extract of *P. tetrastromatica*. The authors associated such a high activity of the extract with the high content of phenolic compounds having strong antibacterial effects (phlorotannin, eckol, and eckol-related compounds) in extracts [62,68]. The authors recommended ethyl acetate extracts from the alga *P. tetrastromatica* as an antibacterial agent for the treatment of infections caused by MRSA. It should be noted that the rest of the extracts also exhibit a rather high antibacterial activity against MRSA.

Various phlorotannins are isolated from extracts, and their biological activity is investigated. Thus, S.H. Eom et al. [66] isolated six phlorotannins from an extract of the brown alga *E. bicyclis* and tested the sensitivity of MRSA to them. The MIC of the compounds ranges from 32 to 64 μg/mL. The highest activity is recorded for phlorofucofuroeckol A (PFF-A). The authors noted a synergistic action of this compound with β-lactam antibiotics, ampicillin, penicillin, and oxacillin.

Oligomeric phenols and eight new natural products, referred to as cladophorols, are obtained from the green alga *C. socialis*. Significantly fewer phenolic compounds are found in this alga than in other species. The compounds are identified using a combination of NMR spectroscopy, mass spectrometry analysis, and computer simulation. Cladophorol C exhibits strong antibacterial activity against MRSA with a MIC of 1.4 μg/mL (control is vancomycin with a MIC of 4 μg/mL). Other cladophorols have lower activity [69,70].

The data presented indicate a promising trend in the development of novel antibacterial agents against drug-resistant *S. aureus* strains based on PTs from a marine alga.

As for the mechanism of the antimicrobial effect of PTs on MRSA, this property of compounds is mediated through the *MEC* operon, which is a part of the *MEC* cassette chromosome (*ScCmec*). MRSA is resistant to β-lactam antibiotics due to its *mecA* gene encoding penicillin-binding protein 2a (PBP2a), whose presence determines a lower affinity for binding β-lactams (penicillins, cephalosporins, and carbopenems) [71]. This protein provides resistance to all β-lactam antibiotics and prevents their clinical use for the treatment of infections caused by MRSA.

PT from the alga *E. bicyclis* is reported to exhibit strong anti-MRSA activity [72]. In a later work, the authors explained the mechanism of this action. The effect of PTs on the expression of staphylococcal genes (*mecA*, *mecI,* and *mecR1*) has been investigated. PT is obtained by extraction from the alga *E. bicycles* with methanol. The methanol extract, in turn, is fractionated with the following solvents: H-hexane, dichloromethane, ethyl acetate, and N-butanol. Earlier, six compounds are isolated from an ethyl acetate extract, among which PFF-A is the most active against MRSA. The MIC of PFF-A against MRSA is 32 μg/mL [66].

The activity of the compounds against MRSA is determined as the number of viable cells of the strain after 24 h of incubation with PFF-A. The integrity of the bacterial cell membrane is assessed by measuring the released cellular material absorbed at 260 nm. PFF-A inhibits cell growth in a dose-dependent manner. At a MIC of 64 μg/mL, cells are completely destroyed. The intracellular contents are extruded. In control, cells, on the contrary, have normal morphology and are viable [66,72].

The authors answered one of the main questions: whether the interaction of PFF-A with bacterial membrane correlates with the antibacterial activity of the compound. For this, the release of intracellular components is measured after adding PFF-A to the culture. Experiments show that the period of adsorption of suspensions at 260 nm significantly increases (up to 40 min) when they are treated with different concentrations of PFF-A. The rate of release of intracellular components depends on the dose and time of exposure to PT. Under the effect of PT, methicillin-resistant staphylococci die within about 40 min. The antibacterial effect of PFF-A is confirmed by the leakage of electrolytes from staphylococci cells exposed to PT [73].

Thus, the results of studies by scientists from different countries allow us to hope that PF of seaweed can be used to create drugs against antibiotic-resistant bacteria, including *S. aureus*, which currently claims thousands of lives in all regions of the world [66,73]. Understanding the genomic mechanisms of action of pharmacologically active metabolites, new possibilities for their preparation and purification, as well as more advanced methods for determining the structure of compounds should open up new possibilities for the practical use of these most interesting and promising biopolymers.

However, as with all areas of drug development, extensive preclinical and clinical trials will be required, with the evidence that in vitro results provide similar in vivo efficacy. In addition, the problem of reproducibility of research results related to natural compounds from batch to batch creates great difficulties since many of the factors described above affect their structure and biological properties. In our opinion, the process can be accelerated by a multidisciplinary strategy—combining the latest advances in different branches of science to solve the important problem for the medicine of overcoming multidrug resistance to drugs of highly virulent pathogens of human and animal diseases.

## 6. Synergism of Marine Algae Phlorotannins and Antibiotics

Antimicrobial resistance of microorganisms is one of the most important public health problems of the 21st century [1,2,3]. Given the widespread occurrence of *S. aureus* in nature and its etiological relationship with serious human infectious diseases, the emergence of multi-resistant strains of this pathogen is a serious threat to society. In addition, *S. aureus* biofilms pose a real risk for in-hospital infection of patients, environmental contamination, or food contamination. Biofilms, which are highly organized communities of bacteria, prevent antibiotics from entering bacteria and protect bacteria from the host’s immune response [74,75].

In this context, seaweed is a very attractive, inexhaustible, and renewable biotechnological resource of polyphenols, with their high antibacterial and antibiofilm potential. A wide range of antimicrobial mechanisms of these natural biopolymers makes it possible to consider them in the near future as a real alternative to traditional antibiotics when used alone or as synergists or enhancers of their action [76,77].

One of the features of the antimicrobial action of polyphenols from algae is a very wide range of potential intracellular targets, including the structural and functional systems of bacteria. It is widely known that polyphenols of plant origin may act as antimicrobial agents with low toxic effects [76,78,79,80,81]. An individual phenolic component could be effective enough to exhibit antibacterial activity, but it may alter its property and behavior in the extract due to the presence of other compounds [82,83,84,85].

In addition to antibacterial studies of natural products, new antimicrobial combinations containing natural product combinations have become a research priority of considerable interest [84,86,87]. The synergy of naturally occurring substances with antibiotics can be an effective strategy for enhancing or restoring antibiotics that are currently ineffective for diseases caused by multi-resistant bacteria, including MRSA. It has been found that phenolic compounds have synergistic effects with some antibiotics [85,88,89].

Various combinations of an antibiotic with polyphenols can enhance or facilitate its interaction with a target in a bacterial cell. At the same time, natural biopolymers act on bacteria using other than antibacterial drugs. In addition, using synergistic substances, it is possible to reduce the toxicity of the antibiotic by reducing the working dose of the drug [82,88,90,91,92].

As shown earlier [87,93], algal PP changes in permeability and integrity of the cell membrane and cell wall, which can facilitate the penetration of antibiotics into the cytoplasm of bacteria. After that, the mechanism of destruction of the pathogen will be carried out by the action of the antibiotic on the vital functions of the microbe—replication, transcription, or translation of DNA, depending on the type of antibiotic [93].

The combined use of phlorotannins and medicinal antimicrobial drugs is a promising approach. In their work, D-S. Lee et al. [94] established a strong synergistic effect of purified dieckol from the alga *E. stolonifera* with commercial β-lactam antibiotics towards methicillin-sensitive and methicillin-resistant *S. aureus*. The interaction of dieckol and β-lactams against MRSA is assessed by the checkerboard method with the determination of fractional inhibitory concentration (FIC index) [94].

In another study, the MIC of ampicillin when acting against two standard MRSA strains sharply decrease (from 512 to 0.5 μg/mL) when the antibiotic is used together with diecol (16 μg). The authors tested the synergism of diekol with penicillin. The result is also a decrease in the MIC of the antibiotic and the establishment of a pronounced synergistic effect against all studied MRSA strains [95].

As studied by Choi et al. [96], eckol obtained from an ethyl acetate extract of the brown alga *E. cava* has an antibacterial effect on MRSA (MIC from 125 to 259 μg/mL). The inhibitory concentration index of the eckol fraction, in combination with ampicillin, ranges from 0.31 to 0.5, which indicates a significant synergism against *S. aureus*.

Clinical trials and in vivo experiments are indicative of the true potential of compounds and the limitations that must be overcome in the development of a dosage form. With regard to pure phlorotannins, unfortunately, so far, studies of the antibacterial action of these compounds are carried out in vitro, but the results obtained by the authors, to date, give hope for the expansion of research. Given the importance of the problem, it seems that in the coming years, serious shifts in this direction are coming.

## 7. Antibiofilm Effects of Polyphenols from Algae

Modern medicine currently faces the threat of not only bacterial drug resistance but also the phenomenon of recurrent infections caused by the formation of bacterial biofilms in tissues of patients damaged by various previous pathological states [97]. Biofilms are associated with 65–80% of all bacterial infections worldwide, thus, posing a major and still difficult-to-resolve healthcare problem.

The marine environment facilitates the formation of microbial biofilms on all abiotic and biotic surfaces underwater [98]. Algae, aquatic organisms carrying out photosynthesis, have to keep their surface free of fouling [99]. For this, they have developed a variety of strategies aimed at preventing the aggregation and colonization of undesirable, including pathogenic microorganisms. These strategies include the formation and the release of specific biologically active anti-fouling agents. These include diterpenoids, volatile compounds, fucoidans, phlorotannins, fucoxanthins, etc., many of which show antibiotic activity against bacteria, fungi, viruses.

Biofilms are microbial communities covered with a common glycocalyx, which is a complex polymeric (polysaccharide) structure. In humans and animals, such biofilms are able to avoid the factors of innate and adaptive immunity [98,100]. They are characterized by an increased rate of horizontal genetic transfer, leading to the acquisition and spread of antibiotic and multidrug resistance. Reproduction foci periodically appear in some zones of biofilm, from which free (planktonic) microorganisms, preserving the phenotype of the original biofilm, are released into the environment. While being within a biofilm, microorganisms have a higher degree of protection against adverse environmental conditions, antimicrobial drugs, and host organism’s immune defense [99,101,102,103]. The problem of bacterial biofilm formation on synthetic implants requires special attention. The formation of bacterial biofilms is accompanied by inflammatory diseases in the oral cavity [104,105]. All these circumstances necessitate a search for alternative antibiotic agents to control microbial communities.

The antibiofilm properties of polyphenolic compounds from terrestrial plants are reported by a significant number of authors [104,106,107,108].

PTs from marine algae have the ability to inhibit the formation of biofilms. M. Puspita [46] showed that enzymatic polyphenolic extracts from the brown alga *Sargassum muticum* have an antibiofilm effect but exhibit no antibacterial effects. *Escherichia coli*, *Pseudomonas aeruginosa*, and *Bacillus subtilis* are selected as biofilm-forming microorganisms. The author explained this contradictory result by the different mechanisms of antibacterial and antibiofilm action. According to the author, it is probably the sugar content in the enzymatic hydrolysate that promotes the growth of bacteria.

Foodborne infections are a major public healthcare issue that causes great economic damage [84].

Serious outbreaks of foodborne infections are caused by food contaminated with *E. coli,* which produces Shiga toxin [109,110]. An example is serotypes of *E. coli* O113:H21 and O154:H10, which form biofilms on surfaces at sites of contact with food [111,112], thus, contaminating it and causing diseases in humans [113,114].

E.W. Bumunang et al. [88] investigated the antibacterial and antibiofilm effects of PT from the brown alga *Ascophyllum nodosum* against two Shiga toxin-producing strains of *E. coli* (serotypes O113:H21 and O154:H10). It has been found that PT could inhibit the biofilm formation by both strains within 24 h of incubation. During this period, the growth of planktonic microorganisms is also inhibited. By 72 h, both strains overcome the inhibitory effect of PT, and the biofilm parameters approach the control values (6.4 log^10^ CFU/cm^2^ in the samples treated with PT; 6.2 log^10^ CFU/cm^2^ in control). A return to the values of the control culture after 72 h is also noted for planktonic microorganisms [88].

Thus, PT stops the process of biofilm formation by *E. coli*. The mechanism of this phenomenon is not completely clear [115]. According to the authors, this possibly occurs through the inhibition of cell growth and the synthesis of exopolysaccharides. In addition, the anti-biofilm effect of PT results from its antiadhesive activity and suppression of quorum sensing (QS) [108,111]. All the above facts may explain the absence of biofilm after 24 h. However, PT suppresses but does not completely stop the growth of bacteria. As this process continues, bacteria reach a sufficient number to inactivate the PT effect and resume biofilm formation subsequently [111,115].

Since it is known that methanol extracts of brown algae mainly consist of phenolic compounds, we consider it relevant to mention very interesting results of the study by A. Busetti et al. [98] in the present review. The authors obtained a methanol extract of the brown alga *Halidrys siliquosa* (E3 extract).

Various strains of *S. aureus* are used for the study. Planktonic cultures of *S. aureus* are sensitive to the E3 extract with MIC and MBC values ranging from 0.1562 to 0.3125 mg/mL. Biofilms of *S. aureus* MRSA 33593 and *S. aureus* MRSA 10442 are sensitive to E3 with MBC values of 1.25 mg/mL and 5 mg/mL, respectively. Planktonic cultures of *Staphylococcus epidermidis* (four strains) are also sensitive to E3; however, mature biofilms of all strains of this microorganism are resistant to the extract. Fluorescence microscopy of 24 h control biofilms that have not been treated with the extract shows their thickness of approximately 7–10 µm and viable bacteria in them (green in color). A different pattern is observed in the preparations to which the E3 extract has been added. There is extensive cell mortality (red in color), which confirms the antibiotic properties of the extract. X-ray analysis of the biofilms shows effective penetration of the extract into all layers of the exopolysaccharide matrix of biofilms [108,111,116].

A different role of algal polyphenols in the destruction of biofilms was described by Z. Tamanai-Shakoori et al. [99]. The authors obtained a polyphenol-rich extract from the alga *A. nodosum*. For the treatment of inflammatory periodontal diseases, a complex is created that inhibits the formation of bacterial biofilms (silver and zeolite) and modulates the inflammatory response of mucous membrane and the oxidative stress due to the presence of polyphenolic compounds. To create this preparation, the authors used ionic silver immobilized on zeolite (a porous crystalline material made of hydrated sodium aluminosilicate with a strong affinity for ionic heavy metals). Two etiological agents of inflammatory diseases of the oral cavity, *Porphyromonas gingivalis* and *Streptococcus gordonii*, are used as biofilm-forming microorganisms. The Ag-zeolite-PP complex is ineffective against the growth of *S. gordonii* but has a pronounced bactericidal effect on *P. gingivalis*. The complex completely inhibits the biofilm formation by *S. gordonii* and significantly reduces in size the biofilm formed by these two microorganisms. The phenolic extract exhibits no bactericidal and antibiofilm activity, but significantly reduces the secretion of tumor necrosis factor alpha (TNFα) and interleukin 6 (IL-6) inflammatory cytokines in lipopolysaccharide-stimulated macrophages and decreases the level of lipid peroxidation in gingival epithelial cells. Such a complex can have antibacterial, antibiofilm, and anti-inflammatory effects in the treatment of diseases of the oral cavity [99].

Thus, polyphenolic compounds from algae show antibiofilm activity. Studies in this field are scarce. However, the available information gives us hope that seaweed-derived PTs will be used to develop methods for controlling multicellular associations of bacteria.

## 8. Prospects for the Use of Polyphenols from Marine Algae in Medical Cosmetology

Acne associated with *Propionibacterium acnes* poses a significant problem, especially among adolescents. It is a chronic inflammatory condition with clinical manifestations ranging from a mild comedonal form to severe cystic acne. The pathogenesis of the disease is multifactorial and complex. *P. acnes* is a key factor in the development of the disease—a Gram-positive aerobic microorganism capable of metabolizing sebaceous triglycerides into fatty acids that attract neutrophils [117]. The development of the disease can be accompanied by disorders of internal organs, as well as a state of hypersensitivity, vomiting, diarrhea, pain in the mouth, and skin redness [118].

In recent years, the antibiotic-resistant *P. acnes* has been increasingly isolated from patients. In this regard, alternative methods for treating acne are of great interest. In particular, methanol extracts from 13 seaweed species in a study by J.S. Choi et al. [96] show inhibitory activity against *P. acnes*, while four of them (from the algae *E. cava*, *Ecklonia curome*, *Ishige sinicola,* and *Symphyocladia latucula*) have a rather strong inhibitory effect, more expressed in the first two species. The zone of suppression of *P. acnes* growth around the disc is 6.3 ± 0.8 mm for *I. sinicola*, 8.8 ± 0.8 mm for *S. laticula*, and 5.3 ± 0.3 mm for *E. cava*. The zone of bacterial growth inhibition around the erythromycin disc, used as a positive control, is 13 mm. The extracts of these algae have no toxic effect on the RAW 264.7 cell line. An important property of the above extracts is their anti-inflammatory effect. Numerous publications indicate that almost all algae extracts and polyphenols, as well as their secondary metabolites, have an anti-inflammatory effect [118,119]. Of particular interest is the fact that the extracts exhibit a bacteriostatic activity not only against *P. acnes* but also against *S. aureus* and *S. epidermidis*, which often accompany acne [120].

Acne is an inflammatory process in which keratinocytes of the skin express pro-inflammatory cytokines and chemokines. S.H. Eom et al. [106], in their experiments on a culture of human skin keratinocytes infected with *P. acnes*, found that eckol from the alga *E. bicyclis* inhibits the expression of proinflammatory cytokines and chemokines, iNOS (NO-synthase), cyclooxygenase-2, as well as the production of NO (nitric oxide), MMP-2 (matrix metalloproteinase-2, also known as type IV collagenase 72 kDa), and MMP-9 (matrix metalloproteinase-9, also known as type IV collagenase 92 kDa). Eckol dose-dependently inhibits phosphorylation, mediated by *P. acnes*, and NF-kB (nuclear factor) activation, which indicates a complex pattern of the action of eckol in acne, which includes, in addition to antibacterial, also anti-inflammatory effects.

In another work, researchers [107] have isolated compounds active against *P. acnes* from methanol extracts. For this, methanol extracts are fractionated by column chromatography on silica gel using solvents with increasing polarities: hexane, diethyl ether, acetone, ethyl acetate, acetonitrile, methanol, and water.

The highest activity against *P. acnes* is exhibited by the acetone fraction, which is additionally purified by column chromatography on gel Sephadex LH-20. Fraction 66–75 shows the highest antibacterial activity. It, in turn, is separated into two fractions identified as dieckol and phlorofucofuroeckol A. The MIC of both fractions is 39 μg/mL. The dieckol exhibits a fungicidal activity (with a MIC of 148 μg/mL) against the fungus *Trichophyton rubrum*, the causative agent of dermatophytic nail infections in humans [107]. These compounds make up 9.2 × 10^−4^% and 6.4 × 10^−4^% of the methanol extract. The authors recommended these compounds as a promising basis for the development of cosmetics and pharmaceuticals.

Other researchers [100] have investigated the antibacterial activity of EtOAc, a soluble fraction of a methanol extract, which is characterized by the following parameters: for *P. acnes*, the growth inhibition zone is 9 mm at a fraction concentration of 1 mg/disk and 12–19 mm at 5 mg/disk; for *S. aureus* and *S. epidermidis*, 10 mm at a fraction concentration of 1 mg/disc and 16 and 20 mm, respectively, at a fraction concentration of 5 mg/disc. At a fraction concentration of 128 and 256 μg/mL, it completely inhibits *P. acnes*. The Gram-negative bacterium *P. aeruginosa* is insensitive to the action of the EtOAc-soluble fraction.

Then, the authors isolated and characterized six phloroglucinol derivatives from the EtOAc-soluble extract of the alga *E. bicyclis*. With respect to *P. acnes*, the MIC of these PTs is 32–64 μg/mL, which proves to be approximately two times lower than the MIC of tetracycline, erythromycin, and lincomycin. One of the compounds shows the best result, from 32 to 128 μg/mL; the other compounds, from 64 to 256 μg/mL [100].

When PP is combined with tetracycline, the MIC of the antibiotic against *P. acnes* decreases from 16 to 8 μg/mL at a PT dose of 64 μg/mL. This indicates that PT from *E. bicyclis* has a weak synergistic effect when used with antibiotics [100,106].

Thus, seaweed-derived phlorotannins are promising alternatives to natural antibiotics and candidates for the development of agents against acne and nutritional supplements as functional food ingredients.

## 9. Importance of Marine Algal Polyphenols as Food Preservatives

Many pathogenic microorganisms cause spoilage of food products, reduce their shelf-life, and cause food poisoning, which affects up to 30% of the population in industrialized countries annually [121]. The chemical preservatives that are currently used in the food industry can, in some cases, result in liver disorders and be mutagenic or neurotoxic. In this regard, antioxidants and antimicrobial preservatives of natural origin are more preferred.

As studies of many researchers have shown, polyphenols act as free-radical scavengers and antimicrobial agents. Thus, Estevinho et al. [122] investigated the antibacterial activity of methanol extracts from 10 polyphenol-rich algae. The highest activity against *S. aureus* is found in extracts from algae *Haligra* sp. taken at a dose of 50 mg/mL. Of particular interest is the fact that the antibacterial activity of the extracts is higher than that of a standard preservative, sodium benzoate (200 mg/mL). The total phenol content, expressed in terms of gallic acid equivalents, in the extracts of *Gelidiella acerosa* and *Haligra* sp. is 0.440 ± 0.0043 g/g and 0.616 ± 0.0063 g/g, respectively. The authors recommended these extracts as an antibacterial preparation against *S. aureus*, a common causative agent of foodborne toxicoinfections that are accompanied by vomiting, diarrhea, and intestinal spasms [122]. This bacterium also causes spoilage of meat, including poultry, dairy products, salads, shrimp, and ham [123]. The extracts have not only antibacterial but also antioxidant effects, suggesting their use as an effective food preservative in an appropriate combination.

*Listeria monocytogenes* is a common causative agent of diseases of various severities associated with the consumption of contaminated foods. This bacterium often appears resistant to antibiotics and disinfectants and is capable of forming biofilms on food [124].

H.J. Kim et al. [123] established the anti-listerial effect of phlorotannins isolated from the alga *E. bicyclis*. Extraction and purification of compounds are carried out, as described. The antibacterial effect of PT is determined by the methods of paper disks and serial dilutions. The growth inhibition zone around methanol extracts is from 9 ± 0.5 mm to 14 ± 0.1 mm. Fucofuroeckol-A exhibits the highest antibacterial activity against *L. monocytogenes* (MIC range 16–32 μg/mL). Chlorofucofuroeckol (MIC 32–128 μg/mL) and dieckol (64–128 μg/mL) have a somewhat weaker effect.

A combination of fucofuroeckol-A with streptomycin provides a synergistic effect against all the *Listeria* strains used in the experiment. The concentration index of the inhibiting fraction is 0.18–0.26–0.53–0.56. The average value is from 0.32 to 0.37, i.e., there is a pronounced synergistic effect. The authors suggested that marine algae could be a source of new effective and low-toxic natural compounds for controlling the spread of *L. monocytogenes* in foods and in the human organism [123].

Thus, although the exact mechanisms of the antimicrobial action of PPs in algae have not yet been fully understood, it is currently known that these promising compounds have different targets for their action in microorganisms that cause food spoilage. In this regard, after careful study, they can be used in the food industry as food preservatives, as well as in protective food films, packaging materials, etc. to increase the shelf life of food.

The possibility of using PPs as antioxidants with antimicrobial properties is an important area, with the aim of commercializing the results of scientific research. In addition, PPs are a necessary component of a healthy diet, and adding these compounds to foods will not only improve their quality and prevent spoilage but also increase their nutritional value. However, further in-depth studies of the PPs of algae suitable for use in food and feed, their safety for humans and animals, and their effectiveness as preservatives are needed.

## 10. Conclusions

Marine brown algae occupy a special position in aquaculture biotopes and are considered an inexhaustible source of unique biologically active substances with a wide range of biomedical properties. The antibacterial activity of marine biopolymers is currently of increasing interest and is actively used in medicine, agriculture, as well as in the food and makeup industries.

In this large group of natural polymers, phlorotannins exhibit particularly attractive antimicrobial properties. Extracts from brown algae containing these polyphenolic metabolites are non-toxic to eukaryotic cells and, at the same time, have a pronounced bactericidal or bacteriostatic effect on a wide range of pathogenic microorganisms, including MRSA.

The high potential for the practical application of phlorotannins as therapeutic and preventive agents is explained by the variety of types of these biopolymers and the multifunctional properties, including also their previously identified potent antioxidant, antiviral, antithrombotic, fungicidal, neuroprotective, and antitumor activities.

The further study of mechanisms of the antimicrobial effect of phlorotannins is associated with the standardization of conditions and methods of extraction of these substances, taking into account regional and seasonal features of their structure and degree of polymerization. The identification of active components, characterization of molecular properties of this group of compounds, and also their biotechnological and pharmacological attractiveness will depend on finding the efficient solution of these issues.

Currently, the mechanisms of the antimicrobial action of polyphenolic compounds from seaweeds are actively studied in different regions of the world, and the features of polyphenol composition and structure are analyzed. Such issues require deep research and accumulation of knowledge since the biological properties of these compounds, associated with the structure of the final products, are determined by the efficiency of extraction methods applied.

When isolating phlorotannins from algal biomass, the thermal instability of the components being isolated, their chemical reactivity, and easy oxidizability, and, on the other hand, the requirements of completeness of extraction, selectivity, and purity of the resulting product should always be taken into account. Therefore, the problem of efficient use of phlorotannins can be resolved by designing comprehensive schemes for the separation of biomass and selective isolation of the target product based on the principles of “environmentally friendly chemistry” and using both classical extraction methods and modern techniques.

The use of modern methods for the fractionation of polyphenolic components by molecular weights is of great importance for this purpose, as it allows not only analysis of the polymer composition of phlorotannin fractions but also the isolation of fractions with desired biological properties [118,121]. The innovative methods for identification, quantification, and structural analysis of phlorotannins are based on nuclear magnetic resonance spectroscopy and chromatography-mass spectrometry with various ionization techniques applied. These analytical tools open up a wide range of opportunities for obtaining, studying the structural diversity of these interesting compounds, and using their most active components to create new pharmaceutical products [92].

Adequate in vivo application of data obtained in vitro requires a careful approach in order to determine the actual antibacterial action of algae-derived polyphenols, as almost all studies on “marine antibiotics” to date have been carried out outside organism. In vivo studies of their effect on effector cells have a number of limitations. The major limitation is the discrepancy between the concentration of drugs used in vitro and those affecting cells of organisms during experimental infections and, moreover, in the conditions of the human body. In in vitro experiments, the internal environment of the organism has almost never been modeled, and the most important serum factors (the complement system, immunoglobulins, etc.) have been ignored. An in vitro study of biologically active substances does not take into account the possible effect of metabolites formed under the conditions of the whole organism on the immune system.

To date, there have been different opinions on the mechanisms of action of various chemical compounds with antibacterial properties extracted from algae. The interaction of algal polyphenols with Gram-positive and Gram-negative bacteria, as well as changes in the ultrastructural organization of bacteria after exposure to polyphenols of different structures, should be studied at the modern methodological level to understand more in detail the role of their antioxidant properties in the antibacterial effect. So far, there are few such works in the literature. The question of targets for polyphenols in bacterial cells (suppression of cell wall synthesis, protein synthesis, nucleic acid synthesis, or other mechanisms) requires clarification through a dedicated study.

Despite all the unresolved issues above, polyphenol compounds from brown algae are considered a promising basis to design new antimicrobial drugs for the treatment and prevention of infectious diseases.

## References

[1] WHO Infectious Disease Newsletter. https://www.who.int/topics/infectious_diseases/factsheets/ru/.

[2] Amorim R.D.N.D.S., Rodrigues J.A.G., Holanda M., Quinderé A.L.G., De Paula R.C.M., Melo V.M.M., Benevides N.M.B. (2012). Antimicrobial effect of a crude sulfated polysaccharide from the red seaweed Gracilaria ornata. Braz. Arch. Biol. Technol..

[3] Zhang W., Oda T., Yu Q., Jin J.-O. (2015). Fucoidan from Macrocystis pyrifera Has Powerful Immune-Modulatory Effects Compared to Three Other Fucoidans. Mar. Drugs.

[4] Li C., Blencke H.-M., Haug T., Jørgensen Ø., Stensvåg K. (2014). Expression of antimicrobial peptides in coelomocytes and embryos of the green sea urchin (*Strongylocentrotus droebachiensis*). Dev. Comp. Immunol..

[5] Van Alstyne K. (1995). Comparison of three methods for quantifying brown algal polyphenolic compounds. J. Chem. Ecol..

[6] Poole J., Diop A., Rainville L.-C., Barnabe S. (2019). Bioextracting Polyphenols from the Brown Seaweed Ascophyllum nodosum from Québec’s North Shore Coastline. Ind. Biotechnol..

[7] Catarino M.D., Silva A.M.S., Mateus N., Cardoso S.M. (2019). Optimization of Phlorotannins Extraction from Fucus vesiculosus and Evaluation of Their Potential to Prevent Metabolic Disorders. Mar. Drugs.

[8] Manandhar B., Paudel P., Seong S.H., Jung H.A., Choi J.S. (2019). Characterizing Eckol as a Therapeutic Aid: A Systematic Review. Mar. Drugs.

[9] Fraga C.G., Croft K.D., Kennedy D.O., Tomás-Barberán F.A. (2019). The effects of polyphenols and other bioactives on human health. Food Funct..

[10] Machů L., Mišurcová L., Ambrozova J.V., Orsavová J., Mlcek J., Sochor J., Jurikova T. (2015). Phenolic Content and Antioxidant Capacity in Algal Food Products. Molecules.

[11] Imbs T.I., Zvyagintseva T.N. (2018). Phlorotannins are polyphenolic metabolites of brown algae. Russ. J. Mar. Biol..

[12] Heffernan N., Brunton N., Fitzgerald R.J., Smyth T.J. (2015). Profiling of the Molecular Weight and Structural Isomer Abundance of Macroalgae-Derived Phlorotannins. Mar. Drugs.

[13] Singh I.P., Sidana J., Bharate S.S., Foley W.J. (2010). Phloroglucinol compounds of natural origin: Synthetic aspects. Nat. Prod. Rep..

[14] Clayton M.N., Lüder U.H. (2004). Induction of phlorotannins in the brown macroalga *Ecklonia radiata* (Laminariales, Phaeophyta) in response to simulated herbivory?the first microscopic study. Planta.

[15] Holdt S.L., Kraan S. (2011). Bioactive compounds in seaweed: Functional food applications and legislation. J. Appl. Phycol..

[16] Sathya R., Kanaga N., Sankar P., Jeeva S. (2017). Antioxidant properties of phlorotannins from brown seaweed *Cystoseira trinodis* (Forsskål) C. Agardh. Arab. J. Chem..

[17] Pérez M.J., Falque E., Domínguez H. (2016). Antimicrobial Action of Compounds from Marine Seaweed. Mar. Drugs.

[18] Jegan S., Raj A., Chandrasekaran M., Vencatesalu V. (2019). Anti-MRSA activity of *Padina tetrastromatica*, *Padina gymnospora* from guft of mannar biosphere. World Sci. News.

[19] Farvin K.S., Jacobsen C. (2013). Phenolic compounds and antioxidant activities of selected species of seaweeds from Danish coast. Food Chem..

[20] Kim S.M., Kang S.W., Jeon J.S., Jung Y.J., Chul W.R.K., Kim Y., Um B.H. (2013). Determination of major phlorotannins in E. bicyclis using hydrophilic interaction chromatography: Seasonal variation and extraction characteristics. Food Chem..

[21] Moon C., Kim S.-H., Kim J.-C., Hyun J.W., Lee N.H., Park J.W., Shin T. (2008). Protective effect of phlorotannin components phloroglucinol and eckol on radiation-induced intestinal injury in mice. Phytother. Res..

[22] Suleria H.A.R., Osborne S.A., Masci P.P., Gobe G.C. (2015). Marine-Based Nutraceuticals: An Innovative Trend in the Food and Supplement Industries. Mar. Drugs.

[23] Koivikko R. (2008). Brown Algal Phlorotannins: Improving and Applying Chemical Methods. Ph.D. Thesis.

[24] Melanson J.E., MacKinnon S.L. (2015). Characterization of Phlorotannins from Brown Algae by LC-HRMS. Adv. Struct. Saf. Stud..

[25] Montero L., Sánchez-Camargo A.P., Garcia-Cañas V., Tanniou A., Stiger-Pouvreau V., Russo M., Rastrelli L., Cifuentes A., Herrero M., Ibáñez E. (2016). Anti-proliferative activity and chemical characterization by comprehensive two-dimensional liquid chromatography coupled to mass spectrometry of phlorotannins from the brown macroalga *Sargassum muticum* collected on North-Atlantic coasts. J. Chromatogr. A.

[26] Clifford M.N. (2004). Diet-Derived Phenols in Plasma and Tissues and their Implications for Health. Planta Med..

[27] Lewandowska U., Szewczyk K., Hrabec E., Janecka A., Gorlach S. (2013). Overview of Metabolism and Bioavailability Enhancement of Polyphenols. J. Agric. Food Chem..

[28] Keyrouz R., Abasq M., Le Bourvellec C., Blanc N., Audibert L., Argall E., Hauchard D. (2011). Total phenolic contents, radical scavenging and cyclic voltammetry of seaweeds from Brittany. Food Chem..

[29] Galleano M., Pechanova O., Fraga C.G. (2010). Hypertension, nitric oxide, oxidants, and dietary plant polyphenols. Curr. Pharm. Biotechnol..

[30] Schoenwaelder M.E.A., Clayton M.N. (1999). The presence of phenolic compounds in isolated cell walls of brown algae. Phycologia.

[31] Lopes G., Sousa C., Silva L.R., Pinto E., Andrade P.B., Bernardo J., Mouga T., Valentão P. (2012). Can Phlorotannins Purified Extracts Constitute a Novel Pharmacological Alternative for Microbial Infections with Associated Inflammatory Conditions?. PLoS ONE.

[32] Gupta S., Abu-Ghannam N. (2011). Recent developments in the application of seaweeds or seaweed extracts as a means for enhancing the safety and quality attributes of foods. Innov. Food Sci. Emerg. Technol..

[33] Chojnacka K., Kim S.-K. (2015). Introduction of Marine Algae Extracts. Mar. Algae Extr. Process. Prod. Appl..

[34] Pádua D., Rocha E., Gargiulo D., Ramos A.A. (2015). Bioactive compounds from brown seaweeds: Phloroglucinol, fucoxanthin and fucoidan as promising therapeutic agents against breast cancer. Phytochem. Lett..

[35] Kim A.-R., Shin T.-S., Lee M.-S., Park J.-Y., Park K.-E., Yoon N.-Y., Kim J.-S., Choi J.S., Jang B.-C., Byun D.-S. (2009). Isolation and Identification of Phlorotannins fromEcklonia stoloniferawith Antioxidant and Anti-inflammatory Properties. J. Agric. Food Chem..

[36] Li Y., Lee S.-H., Le Q.-T., Kim M.-M., Kim S.-K. (2008). Anti-allergic Effects of Phlorotannins on Histamine Release via Binding Inhibition between IgE and Fc epsilonRI. J. Agric. Food Chem..

[37] Ahn M.-J., Yoon K.D., Min S.-Y., Lee J.S., Kim J.H., Kim T.G., Kim S.H., Kim N.-G., Huh H., Kim J. (2004). Inhibition of HIV-1 reverse transcriptase and protease by phlorotannins from the brown alga Ecklonia cava. Biol. Pharm. Bull..

[38] Parys S., Kehraus S., Krick A., Glombitza K.-W., Carmeli S., Klimo K., Gerhauser C., König G.M. (2010). In vitro chemopreventive potential of fucophlorethols from the brown alga *Fucus vesiculosus* L. by anti-oxidant activity and inhibition of selected cytochrome P450 enzymes. Phytochemistry.

[39] Wijesekara I., Yoon N.Y., Kim S.K. (2010). Phlorotannins from Ecklonia cava (Phaeophyceae): Biological activities and potential health benefits. Biofactors..

[40] Wang T., Jónsdóttir R., Liu H., Gu L., Kristinsson H.G., Raghavan S., Olafsdottir G. (2012). Antioxidant Capacities of Phlorotannins Extracted from the Brown Algae *Fucus vesiculosus*. J. Agric. Food Chem..

[41] Audibert L., Fauchon M., Blanc N., Hauchard D., Gall E.A. (2010). Phenolic compounds in the brown seaweed *Ascophyllum nodosum*: Distribution and radical-scavenging activities. Phytochem. Anal..

[42] Shibata T., Ishimaru K., Kawaguchi S., Yoshikawa H., Hama Y. (2007). Antioxidant activities of phlorotannins isolated from Japanese Laminariaceae. Environ. Biol. Fishes.

[43] Aminina N.M., Vishnevskaya T.I., Karaulova E.P., Epur N.V., Yakush E.V. (2020). Prospects for the Use of Commercial and Potentially Commercial Brown Algae of the Far Eastern Seas as a Source of Polyphenols. Russ. J. Mar. Biol..

[44] Aminina N.M., Vishnevskaya T.I., Karaulova E.P., Yakush E.V. (2017). Content of polyphenols and antioxidant activity of extracts from certain species of seaweeds. TINRO News.

[45] Moizer E.B., Skerget M., Knez S., Epur N.V., Yakush E.V. (2016). Polyphenols: Extraction methods, antioxidative action, bioavailability and anticancerogenic effects. Molecules.

[46] Puspita M., Déniel M., Widowati I., Radjasa O.K., Douzenel P., Marty C., Vandanjon L., Bedoux G., Bourgougnon N. (2017). Total phenolic content and biological activities of enzymatic extracts from *Sargassum muticum* (Yendo) Fensholt. Environ. Biol. Fishes.

[47] Rajbhar K., Dawda H., Mucundan U. (2015). Polyphenols: Methods of extraction. Sci. Rev. Chem. Commun..

[48] Li Y., Fu X., Duan D., Liu X., Xu J., Gao X. (2017). Extraction and identification of phlorotannins from the brown alga *Sargassum fusiforme* (Harwey) Setchell. Mar. Drugs.

[49] Ummat V., Tiwari B., Jaiswal A., Condon K., García-Vaquero M., O’Doherty J.V., O’Donnell C., Rajauria G. (2020). Optimisation of Ultrasound Frequency, Extraction Time and Solvent for the Recovery of Polyphenols, Phlorotannins and Associated Antioxidant Activity from Brown Seaweeds. Mar. Drugs.

[50] Stern J.L., Hagerman A.E., Steinberg P.D., Mason P.K. (1996). Phlorotannin-protein interactions. J. Chem. Ecol..

[51] Moubayed N.M., Al Houri H.J., Al Khulaifi M.M., Al Farraj D.A. (2016). Antimicrobial, antioxidant properties and chemical composition of seaweeds collected from Saudi Arabia (Red Sea and Arabian Gulf). Saudi J. Biol. Sci..

[52] Venkatesan J., Keekan K.K., Anil S., Bhatnagar I., Kim S.-K. (2019). Phlorotannins. Encycl. Food Chem..

[53] Heldt H.-W., Piechulla B. (2010). Plant Biochemistry.

[54] Wang Y., Xu Z., Bach S.J., McAllister T.A. (2009). Sensitivity of Escherichia coli to Seaweed (*Ascophyllum nodosum*) Phlorotannins and Terrestrial Tannins. Asian-Australasian J. Anim. Sci..

[55] Golan D.E., Tashjian A.H., Armstrong E.J. (2011). Principles of Pharmacology: The Pathophysiologic Basis of Drug Therapy.

[56] Wei Y., Liu Q., Xu C., Yu J., Zhao L., Guo Q. (2015). Damage to the Membrane Permeability and Cell Death of *Vibrio parahaemolyticus* Caused by Phlorotannins With Low Molecular Weight from *Sargassum thunbergii*. J. Aquat. Food Prod. Technol..

[57] McDonnell G.E., Russell A.D. (1999). Antiseptics and Disinfectants: Activity, Action, and Resistance. Clin. Microbiol. Rev..

[58] McDonnell G.E. (2007). Chemical Disinfection. Antisepsis, Disinfection, and Sterilization: Types, Action, and Resistance.

[59] Chibane L.B., Degraeve P., Ferhout H., Bouajila J., Oulahal N. (2018). Plant antimicrobial polyphenols as potential natural food preservatives. J. Sci. Food Agric..

[60] Hierholtzer A., Chatellard L., Kierans M., Akunna J., Collier P. (2013). The impact and mode of action of phenolic compounds extracted from brown seaweed on mixed anaerobic microbial cultures. J. Appl. Microbiol..

[61] Vissers A.M., Caligiani A., Sforza S., Vincken J.-P., Gruppen H. (2017). Phlorotannin Composition of *Laminaria digitata*. Phytochem. Anal..

[62] Nagayama K., Iwamura Y., Shibata T., Hirayama I., Nakamura T. (2002). Bactericidal activity of phlorotannins from the brown alga *Ecklonia kurome*. J. Antimicrob. Chemother..

[63] Kim E.S., Song J.S., Lee H.J., Choe P.G., Park K.H., Cho J.H., Park W.B., Kim S.-H., Bang J.H., Kim C.-M. (2007). A survey of community-associated methicillin-resistant Staphylococcus aureus in Korea. J. Antimicrob. Chemother..

[64] Kamei Y., Isnansetyo A. (2003). Lysis of methicillin-resistant Staphylococcus aureus by 2,4-diacetylphloroglucinol produced by Pseudomonas sp. AMSN isolated from a marine alga. Int. J. Antimicrob. Agents.

[65] Brumfitt W., Hamilton-Miller J.M.T. (1999). The worldwide problem of methicillin-resistant Staphylococcus aureus. Drugs Exp. Clin. Res..

[66] Eom S.-H., Kim D.-H., Lee S.-H., Yoon N.-Y., Kim J.H., Kim T.H., Chung Y.-H., Kim S.-B., Kim Y.-M., Kim H.-W. (2012). In VitroAntibacterial Activity and Synergistic Antibiotic Effects of Phlorotannins Isolated fromEisenia bicyclisAgainst Methicillin-ResistantStaphylococcus aureus. Phytother. Res..

[67] Liu C., Graber C.J., Karr M., Diep B.A., Basuino L., Schwartz B.S., Enright M.C., O’Hanlon S., Thomas J.C., Perdreau-Remington F. (2008). A Population-Based Study of the Incidence and Molecular Epidemiology of Methicillin-ResistantStaphylococcus aureusDisease in San Francisco, 2004–2005. Clin. Infect. Dis..

[68] Ebrahimzadeh M.A., Khalili M., Dehpour A.A. (2018). Antioxidant activity of ethyl acetate and methanolic extracts of two marine algae, Nannochloropsis oculata and Gracilaria gracilis—An in vitro assay. Braz. J. Pharm. Sci..

[69] Lavoie S., Sweeney-Jones A.M., Mojib N., Dale B., Gagaring K., McNamara C.W., Quave C.L., Soapi K., Kubanek J. (2019). Antibacterial Oligomeric Polyphenols from the Green Alga *Cladophora socialis*. J. Org. Chem..

[70] Worthington R.J., Melander C. (2013). Combination approaches to combat multidrug-resistant bacteria. Trends Biotechnol..

[71] Eom S.-H., Kang Y.-M., Park J.-H., Yu D.-U., Jeong E.-T., Lee M.-S., Kim Y.-M. (2011). Enhancement of Polyphenol Content and Antioxidant Activity of Brown Alga Eisenia bicyclis extract by Microbial Fermentation. Fish. Aquat. Sci..

[72] Eom S.-H., Lee D.-S., Jung Y.-J., Park J.-H., Choi J.-I., Yim M.-J., Jeon J.-M., Kim H.-W., Son K.-T., Je J. (2014). The mechanism of antibacterial activity of phlorofucofuroeckol-A against methicillin-resistant Staphylococcus aureus. Appl. Microbiol. Biotechnol..

[73] Howard S.J., Hopwood S., Davies S.C. (2014). Antimicrobial Resistance: A Global Challenge. Sci. Transl. Med..

[74] Harrison J.J., Ceri H., Turner R.J. (2007). Multimetal resistance and tolerance in microbial biofilms. Nat. Rev. Genet..

[75] Høiby N., Bjarnsholt T., Givskov M., Molin S., Ciofu O. (2010). Antibiotic resistance of bacterial biofilms. Int. J. Antimicrob. Agents.

[76] Maisonneuve E., Gerdes K. (2014). Molecular Mechanisms Underlying Bacterial Persisters. Cell.

[77] Takahashi N., Nyvad B. (2011). The role of bacteria in the caries process: Ecological perspectives. J. Dent. Res..

[78] Marsh P.D., Do T., Devine D. (2013). Oral biofilms: Molecular analysis, challenges, and future prospects in dental diagnostics. Clin. Cosmet. Investig. Dent..

[79] Gutiérrez-Barranquero J.A., Reen F.J., McCarthy R.R., O’Gara F. (2015). Deciphering the role of coumarin as a novel quorum sensing inhibitor suppressing virulence phenotypes in bacterial pathogens. Appl. Microbiol. Biotechnol..

[80] Cho H.S., Lee J.-H., Cho M.H., Lee J. (2014). Red wines and flavonoids diminish Staphylococcus aureus virulence with anti-biofilm and anti-hemolytic activities. Biofouling.

[81] Shen X.-F., Ren L.-B., Teng Y., Zheng S., Yang X.-L., Guo X.-J., Wang X.-Y., Sha K.-H., Li N., Xu G.-Y. (2014). Luteolin decreases the attachment, invasion and cytotoxicity of UPEC in bladder epithelial cells and inhibits UPEC biofilm formation. Food Chem. Toxicol..

[82] Rendeková K., Fialová S., Jánošová L., Mučaji P., Slobodníková L. (2015). The Activity of Cotinus coggygria Scop. Leaves on Staphylococcus aureus Strains in Planktonic and Biofilm Growth Forms. Molelules.

[83] Asahi Y., Noiri Y., Miura J., Maezono H., Yamaguchi M., Yamamoto R., Azakami H., Hayashi M., Ebisu S. (2014). Effects of the tea catechin epigallocatechin gallate on *Porphyromonas gingivalis biofilms*. J. Appl. Microbiol..

[84] Lee P., Tan K.S. (2015). Effects of Epigallocatechin gallate against *Enterococcus faecalis* biofilm and virulence. Arch. Oral Biol..

[85] Hussain M.A., Dawson C.O. (2013). Economic Impact of Food Safety Outbreaks on Food Businesses. Foods.

[86] Mikhail A.F.W., Jenkins C., Dallman T.J., Inns T., Douglas A., Martín A.I.C., Fox A., Cleary P., Elson R., Hawker J. (2018). An outbreak of Shiga Toxin-producing Escherichia coli O157:H7 associated with contaminated salad leaves: Epidemiological, genomic and food trace back investigations. Epidemiol. Infect..

[87] Wilson D., Dolan G., Aird H., Sorrell S., Dallman T.J., Jenkins C., Robertson L., Gorton R. (2018). Farm-to-fork investigation of an outbreak of Shiga toxin-producing *Escherichia coli* O157. Microb. Genom..

[88] Bumunang E.W., McAllister T.A., Zaheer R., Polo R.O., Stanford K., King R., Niu Y.D., Ateba C.N. (2019). Characterization of Non-O157 Escherichia coli from Cattle Faecal Samples in the North-West Province of South Africa. Microorganisms.

[89] Bumunang E.W., Ateba C., Stanford K., McAllister T.A., Niu J.D. (2020). Biofilm formation by South-African Shiga toxigenic non-O157 Escherichia coli on stainless steel coupons. Can. J. Microbiol..

[90] Wang R. (2018). Biofilms and Meat Safety: A Mini-Review. J. Food Prot..

[91] Galiè S., García-Gutiérrez C., Miguélez E.M., Villar C.J., Lombó F. (2018). Biofilms in the Food Industry: Health Aspects and Control Methods. Front. Microbiol..

[92] Lu L., Hu W., Tian Z., Yuan D., Yi G., Zhou Y., Cheng Q., Zhu J., Li M. (2019). Developing natural products as potential anti-biofilm agents. Chin. Med..

[93] Moraes J.O., Cruz E.A., Souza E.G., Oliveira T.C., Alvarenga V.O., Peña W.E., Sant’Ana A.S., Magnani M. (2018). Predicting adhesion and biofilm formation boundaries on stainless steel surfaces by five Salmonella enterica strains belonging to different serovars as a function of pH, temperature and NaCl concentration. Int. J. Food Microbiol..

[94] Lee D.-S., Kang M.-S., Hwang H.-J., Eom S.-H., Yang J.-Y., Lee M.-S., Lee W.-J., Jeon Y.-J., Choi J.-S., Kim Y.-M. (2008). Synergistic effect between dieckol from Ecklonia stolonifera and β-lactams against methicillin-resistant Staphylococcus aureus. Biotechnol. Bioprocess Eng..

[95] Norden C.W., Wentzel H., Keleti E. (1979). Comparison of Techniques for Measurement of in Vitro Antibiotic Synergism. J. Infect. Dis..

[96] Oliver S.P. (2019). Foodborne Pathogens and Disease Special Issue on the National and International PulseNet Network. Foodborne Pathog Dis..

[97] Ford L., Stratakos A.C., Theodoridou K., Dick J.T.A., Sheldrake G.N., Linton M., Corcionivoschi N., Walsh P.J. (2020). Polyphenols from Brown Seaweeds as a Potential Antimicrobial Agent in Animal Feeds. ACS Omega.

[98] Busetti A., Thompson T.P., Tegazzini D., Megaw J., Maggs C.A., Gilmore B.F. (2015). Antibiophilm activity of the brown alga Halidrys siliquosa against clinically relevant human pathogens. Mar. Drugs.

[99] Tamanai-Shacoori Z., Chandal F., Rebillard A., Cillard J., Bonnaure-Mallet M. (2014). Silver-zeolite combided to polyphenol-rich-extracts of Ascophyllum nodosum: Potential active role in prevention of periodontal diseases. PLoS ONE.

[100] Jappe U. (2003). Pathological mechanisms of acne with special emphasis on Propionibacterium acnes and related therapy. Acta Derm. Venereol..

[101] Kim S.S., Baik J.S., Oh T.H., Yoon W.J., Lee N.H., Hyun C.G. (2008). Biological activities of corean Citrus obovoides and Citrus natsudaidai essential oils against acne-inducing bacteria. Biosci. Biotechnol..

[102] Choi J.-S., Bae H.-J., Kim S.-J., Choi I.S. (2011). In vitro antibacterial and anti-inflammatory properties of seaweed extracts against acne inducing bacteria, Propionibacterium acnes. J. Environ. Biol..

[103] Yu Y., Wang L., Fu X., Fu X., Yang M., Han Z., Mou H., Jeon Y.-J., Wang L. (2019). Anti-oxidant and anti-inflammatory activities of ultrasonic-assistant extracted polyphenol-rich compounds from Sargassum muticum. J. Oceanol. Limnol..

[104] Gomez-Guzman M., Rodrigues-Nogales A., Algieri F., Galves J. (2018). Potential role of seaweed polyphenols in cardio-vascular-associated disorders. Mar. Drugs..

[105] Dréno B. (2017). What is new in the pathophysiology of acne, an overview. J. Eur. Acad. Dermatol. Venereol..

[106] Eom S.-H., Lee E.-H., Park K., Kwon J.-Y., Kim P.-H., Jung W.-K., Kim Y.-M. (2016). Eckol fromEisenia bicyclisInhibits Inflammation Through the Akt/NF-κB Signaling inPropionibacterium acnes-Induced Human Keratinocyte Hacat Cells. J. Food Biochem..

[107] Choi J.-S., Lee K., Lee B.-B., Kim Y.C., Kim Y.D., Hong Y.K., Cho K.K., Choi I.S. (2014). Antibacterial activity of the phlorotannins dieckol and phlorofucofuroeckol-A from Ecklonia cava against Propionibacterium acnes. Bot. Sci..

[108] Lee M.H., Lee K.B., Oh S.M., Lee B.H., Chee H.Y. (2010). Anti-fungal activities of dieckol isolated from the marine brown alga Ecklonia cava against Trichophyton rubrum. J. Korean Soc. Appl. Biol. Chem..

[109] Lee J.-H., Eom S.-H., Lee E.-H., Jung Y.-J., Kim H.-J., Jo M.-R., Son K.-T., Lee H.-J., Kim J.H., Lee M.-S. (2014). In vitro antibacterial and synergistic effect of phlorotannins isolated from edible brown seaweed Eisenia bicyclis against acne-related bacteria. Algae.

[110] WHO N.D. (2002). Food Safety and Food Borne Illness. Biochim. Clin..

[111] Prescott L.M., Harley J.P., Klein D.A. (2002). Microbiology 5.

[112] Chen B.-Y., Pyla R., Kim T.-J., Silva J.L., Jung Y.-S. (2010). Prevalence and contamination patterns of Listeria monocytogenes in catfish processing environment and fresh fillets. Food Microbiol..

[113] Eom S.-H., Kim Y.-M., Kim S.-K. (2012). Antimicrobial effect of phlorotannins from marine brown algae. Food Chem. Toxicol..

[114] Xu M., Xue H., Li X., Zhao Y., Lin L., Yang L., Zheng G. (2019). Chemical composition, antibacterial properties, and mechanism of Smilax china L. polyphenols. Appl. Microbiol. Biotechnol..

[115] Bhattacharya D., Ghosh D., Sarkar S., Karmakar P., Koley H., Gachhui R. (2018). Antibacterial activity of polyphenolic fraction of Kombucha against Vibrio cholerae: Targeting cell membrane. Lett. Appl. Microbiol..

[116] Daglia M. (2012). Polyphenols as antimicrobial agents. Curr. Opin. Biotechnol..

[117] Pereira V., Dias C., Vasconcelos M., Rosa E.A., Saavedra M.J. (2014). Antibacterial activity and synergistic effects between *Eucalyptus globulus* leaf residues (essential oils and extracts) and antibiotics against several isolates of respiratory tract infections (*Pseudomonas aeruginosa*). Ind. Crop. Prod..

[118] Ziani B.E., Heleno S.A., Bachari K., Dias M.I., Alves M.J., Barros L., Ferreira I.C. (2019). Phenolic compounds characterization by LC-DAD- ESI/MSn and bioactive properties of *Thymus algeriensis* Boiss. & Reut. and *Ephedra alata* Decne. Food Res. Int..

[119] Vasconcelos N., Croda J., Simionatto S. (2018). Antibacterial mechanisms of cinnamon and its constituents: A review. Microb. Pathog..

[120] Borchers A.T., Keen C.L., Gershwin M.E. (2004). Mushrooms, Tumors, and Immunity: An Update. Exp. Biol. Med..

[121] Devi K.P., Suganthy N., Kesika P., Pandian S.K. (2008). Bioprotective properties of seaweeds: In vitro evaluation of antioxidant activity and antimicrobial activity against food borne bacteria in relation to polyphenolic content. BMC Complement. Altern. Med..

[122] Estevinho L.M., Pereira A., Moreira L.F., Dias L.G., Pereira E.L. (2008). Antioxidant and antimicrobial effects of phenolic compounds extracts of Northeast Portugal honey. Food Chem. Toxicol..

[123] Kim H.-J., Dasagrandhi C., Eom S.-H. (2017). In Vitro Antibacterial Activity of Phlorotannins from Edible Brown Algae, Eisenia bicyclis Against Streptomycin-Resistant Listeria monocytogenes. Indian J. Microbiol..

[124] Lee B.H., Cole S., Mioche L., Badel-Berchoux S. (2019). Biofilm Formation of Listeria monocytogenes Strains Under Food Processing Environments and Pan-Genome-Wide Association Study. Front Microbiol..

